# Autoimmune Myelofibrosis and Systemic Lupus Erythematosus in a Middle-Aged Male Presenting Only with Severe Anemia: A case report

**DOI:** 10.1097/MD.0000000000000741

**Published:** 2015-05-21

**Authors:** Xerxes Pundole, Sergej Konoplev, Thein Hlaing Oo, Huifang Lu

**Affiliations:** From the Section of Rheumatology, Department of General Internal Medicine (XP,HL); Department of Hematopathology, Division of Pathology/Lab Medicine (SK) and Section of Benign Hematology, Division of Internal Medicine, The University of Texas MD Anderson Cancer Center, Houston, Texas, USA (THO).

## Abstract

Autoimmune myelofibrosis is a distinct clinicopathologic entity that occasionally occurs with autoimmune disorders such as systemic lupus erythematosus (SLE) and rheumatoid arthritis. Most cases of autoimmune myelofibrosis have been reported in female patients with a known history of SLE. We report a case of a middle-aged male patient with an unusual presentation of SLE and autoimmune myelofibrosis who presented only with severe anemia initially and was later diagnosed with SLE and autoimmune myelofibrosis. The patient's condition improved dramatically after treatment with corticosteroids.

SLE and autoimmune myelofibrosis is a rare but potentially devastating condition. Anemia maybe the only presenting symptom in addition to bone marrow fibrosis and careful clinical and laboratory assessment is imperative. Corticosteroids maybe useful and spare patients from bone marrow transplantation.

## INTRODUCTION

Patients with systemic lupus erythematosus (SLE) commonly have hematologic abnormalities such as anemia, leukopenia, and thrombocytopenia.^1,2^ SLE rarely causes bone marrow fibrosis, and only a few cases of the coexistence of these conditions have been reported. Myelofibrosis in autoimmune disorders such as SLE is referred to as autoimmune myelofibrosis, which results in isolated or combined chronic cytopenias.^3,4^ Most cases of autoimmune myelofibrosis have been described in young female patients with a known history of SLE.^2–8^ Presented here is a male patient who presented with severe anemia and no known history of SLE, who was later diagnosed with autoimmune myelofibrosis. After this unusual presentation, the patient was treated with corticosteroids and the blood counts improved dramatically, sparing the patient from bone marrow transplantation.

We obtained verbal consent from the patient for use of this case for teaching and publication purposes. Only de-identified data have been used in this case report and institutional review board approval was not obtained.

## CASE REPORT

**FIGURE 1:**
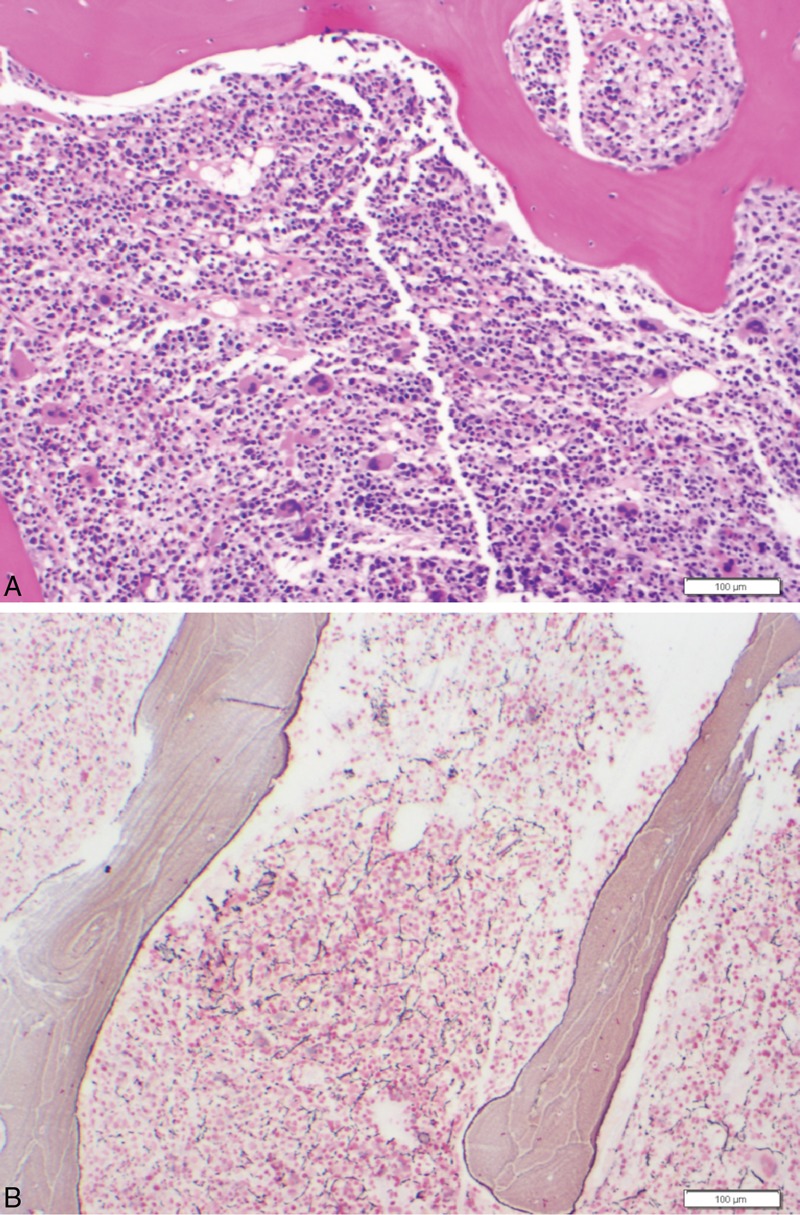
Markedly hypercellular bone marrow with moderate reticulin fibrosis, left shift, and megakaryocytic hyperplasia without overt megakaryocytic dysplasia.

In July 2004, a 41-year-old white male patient presented to his primary care physician with fatigue, weakness, cough, and fever. He denied any rectal bleeding or melena. Initial workup revealed severe anemia: hemoglobin was 5.2 g/dL, the white blood cell count (WBC) was 2700 /μL, and the platelet count was 161,000/μL. A direct Coombs test was positive for immunoglobulin G (IgG) and for complement. Lactate dehydrogenase was normal and plasma haptoglobin was 301 mg/dL (reference range 30–221 mg/dL), thus ruling out hemolysis. A peripheral blood smear showed normocytic normochromic anemia with occasional large platelets. His bone marrow was markedly hypercellular for his age (90%) and showed left shift and megakaryocytes demonstrating very mild atypia (rare left-shifted, small, and hypolobated forms) and moderate reticulin fibrosis, suggestive of a myeloproliferative neoplasm (Figure 1). Of note, the degree of megakaryocyte atypia did not reach the diagnostic criteria for dysmegakaryopoiesis; no megakaryocyte clustering or overt megakaryocytic dysplasia (specifically, no staghorn, cloud-like, naked, or bizarre forms) were detected. A CT scan of the abdomen and pelvis was unremarkable, showing no evidence of splenomegaly. The patient underwent a transfusion with 3 units of packed red cells and was referred to The University of Texas MD Anderson Cancer Center for further evaluation.

A review of systems did not reveal any abnormalities. A repeat bone marrow examination revealed hypercellular marrow with active trilineage hematopoiesis with no evidence of dysplasia or lymphoma. Bone marrow cytogenetic studies revealed 46, XY in 20 of 20 metaphases. Immunostains for Epstein–Barr virus and parvovirus were negative, as was the serum protein electrophoresis. Lactate dehydrogenase was 550 IU/L (reference range 313–618 IU/L), alkaline phosphatase was 71 IU/L (reference range 38–126 IU/L), and serum uric acid was 5.5 mg/dL (reference range 2.6–7.1 mg/dL), all within the normal ranges. An HIV-1 and HIV-2 antibody test was nonreactive. An elevated erythrocyte sedimentation rate (54 mm/hour) and positive antinuclear antibody (ANA) test (≥1:640; normal <1:40) with a speckled pattern were observed, but there was no clinical evidence of joint pain, malar rashes, photosensitivity, or other internal organ involvement to suggest a collagen vascular disease. A double-stranded DNA antibody test was negative. Urine analysis was negative for red blood cells and protein, and electrocardiogram and echocardiogram results were within normal limits. Table 1 shows other pertinent laboratory findings. Given the lack of any other signs or symptoms of SLE or other autoimmune diseases, a working diagnosis of autoimmune myelofibrosis was reached because the patient did not match the typical criteria for primary myelofibrosis. A matched allogeneic stem cell transplantation was planned.

**TABLE 1:**
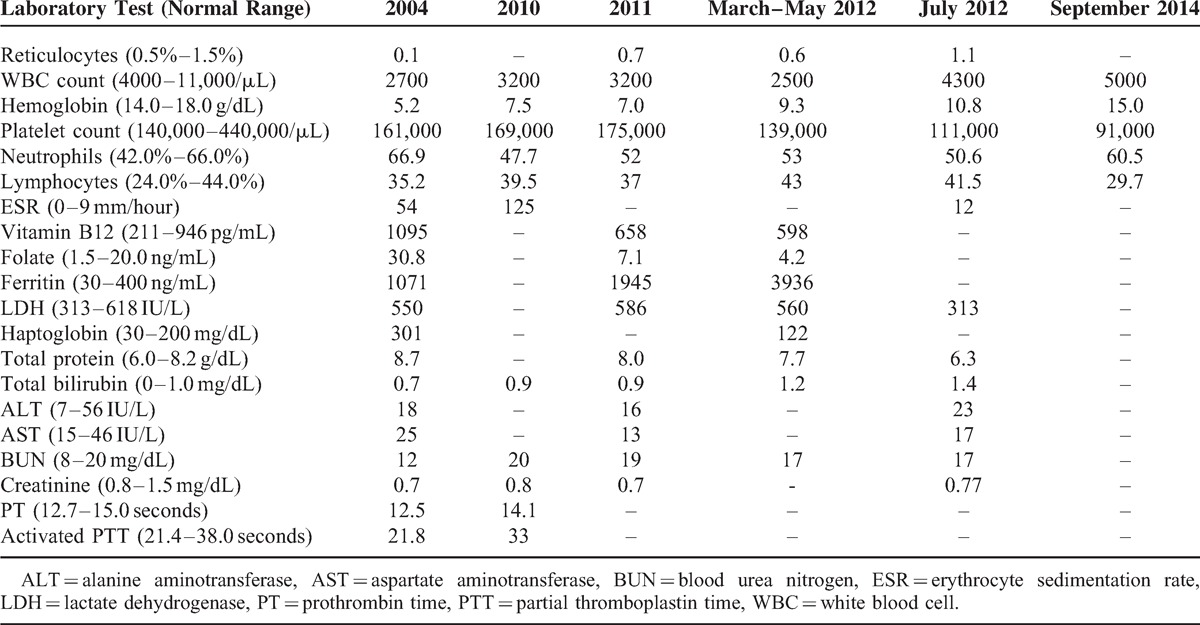
Critical Laboratory Findings Related to Disease Progression and Remission in Our Patient

**FIGURE 2:**
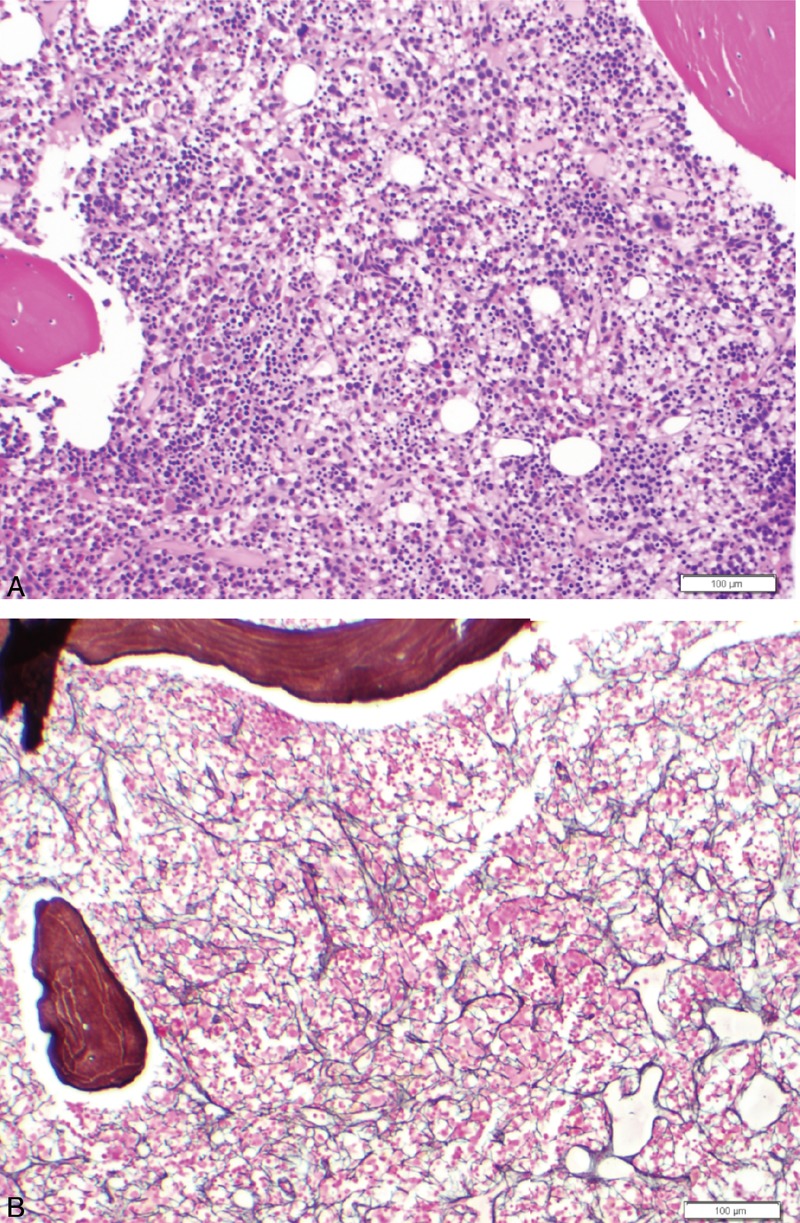
Markedly hypercellular bone marrow with more prominent reticulin fibrosis compared with the previous biopsy, as well as megakaryocytic hyperplasia without overt megakaryocytic dysplasia.

Between July 2004 and June 2005, according to available outside records, the patient required red blood cell transfusions every 4–6 weeks. Thereafter the patient relocated to Colorado. The patient reported that his hemoglobin levels stabilized spontaneously in 2005, and did not undergo the transplantation or require any therapy. However, he returned to MD Anderson in August 2010 for reevaluation when his hemoglobin level dropped to 7–8 g/dL. He again became dependent on transfusions every month and his hemoglobin nadir was approximately 6.5 g/dL. A repeat bone marrow biopsy in March 2011 revealed markedly hypercellular marrow (95%) with more prominent reticulin fibrosis compared with the previous biopsy and with megakaryocytic hyperplasia without overt megakaryocytic dysplasia (Figure 2). An iron stain was performed on the aspirate smear; the stain showed elevated storage iron (4+ on a 0–4+ scale), but no abnormal iron incorporation was noted. JAK-2 mutation analysis was negative. Bone marrow cytogenetics revealed 46, XY in all metaphases.

In early 2012, the patient developed cold-like symptoms with bronchitis, pneumonia, and subsequently a pleural effusion. He was started on 20 mg prednisone orally once a day and antibiotics, which helped with his pleural effusion and cough. He was again evaluated for stem cell transplantation and at the same time was also referred to the rheumatology clinic. He denied ever having oral or nasal ulcerations, alopecia, Raynaud disease, hematuria, hemoptysis, or joint complaints. He had a photosensitive macular rash in his lower extremities that was nonblanching and nonpruritic. He also had splenomegaly, which was evident upon palpation (4 cm below the left costal margin). The reticulocyte count was 0.6%. His ANA test results were positive (1:320; normal <1:40) with a homogenous pattern, and his double-stranded DNA antibody test results were also positive (49.7 IU/mL; negative <30.0). A direct Coombs test was weakly positive for IgG. With the sequential presentation of severe anemia and fatigue, positive ANA test results, pleural effusion, and a photosensitive rash, a diagnosis of SLE was considered. A trial of steroid treatment was administered. The patient received 1 g intravenous methylprednisolone for 3 days followed by 60 mg oral prednisone, which was slowly tapered to 20 mg. For infection prophylaxis, while the patient was receiving high-dose steroids, he also received prophylactic antibiotic (trimethoprim and sulfamethoxazole,1 double strength tablet orally 3 times per week), antifungal (fluconazole 100 mg, 1 tablet orally twice per day), and antiviral (valacyclovir 500 mg, 1 tablet orally twice per day) medication. Infection prophylaxis was discontinued when steroids were tapered.

The patient's overall condition improved. His severe anemia, which was the only manifestation of lupus at the time of initial presentation, completely resolved with the steroid treatment and his platelet count remained around 120,000 to 130,000/μL. His complement levels (C3 and C4) were within normal range. The dose of prednisone was tapered down to <10 mg daily and the patient continued to receive 400 mg hydroxychloroquine daily. In December 2013, his platelet count started to drop to 86,000/μL. Hydroxychloroquine was discontinued because further decline in the platelet count was felt to be a side effect. Despite this, the platelet count continued to remain low. Splenomegaly with mild hypersplenism might be partly responsible for the unresolved thrombocytopenia. A repeat bone marrow biopsy in March 2014 revealed findings similar to the sample from 2011, but as of the writing of this case report, the patient is doing well and has no significant complaints.

## DISCUSSION

Hematologic abnormalities such as anemia, autoimmune hemolysis, aplastic anemia, leukopenia, and thrombocytopenia are frequently observed in patients with SLE.^2^ Myelofibrosis rarely causes cytopenias in SLE; the converse also holds true (ie, SLE rarely causes myelofibrosis).^9^ Myelofibrosis is a prominent feature in hematologic neoplasms but is rarely observed in autoimmune disorders,^8,10,11^ and most of the cases reported have been associated with established SLE and have occurred primarily in female patients.^3,4,8^ Autoimmune myelofibrosis is a term that was introduced by Paquette et al in 1994.^3^ Its clinicopathologic pattern includes reticulin and/or collagen fibrosis, as well as varying degrees of cellularity in the bone marrow (hypocellularity to hypercellularity); the absence of megakaryocytic, myeloid, or erythroid dysplasia; peripheral cytopenias with teardrop poikilocytosis and leukoerythroblastosis; bone marrow lymphoid aggregates; and the presence of autoantibodies.^3,4,8^

In our case, the patient presented with severe anemia. Bone marrow biopsies revealed hypercellular bone marrow (90%) with active trilineage hematopoiesis, atypical megakaryocytic hyperplasia, left-shifted ineffective erythropoiesis, and focal fibrosis, suggestive of a myeloproliferative neoplasm. At the patient's initial presentation the ANA test was positive, but no other signs and symptoms were present to support a diagnosis of SLE or other autoimmune disorder. A working diagnosis of an autoimmune cause for myelofibrosis was reached owing to the lack of the typical features of primary myelofibrosis. Owing to circumstances, the patient did not undergo allogeneic stem cell transplantation and exhibited spontaneous hematologic improvement for approximately 4 years. After relapse, he had a full autoimmune workup and developed a photosensitive rash, pleural effusion, double-stranded DNA antibodies, and anemia, and he was subsequently diagnosed with SLE. The clinical course of this patient is unique in that he did not meet American College of Rheumatology^12^ criteria for a diagnosis of SLE upon initial presentation, but years later had signs and symptoms sequentially suggestive of SLE. Our case is similar to those reported by Bass et al,^10^ in which none of the 3 cases reported met the standard criteria for the diagnosis of SLE. Most cases of autoimmune myelofibrosis described thus far occurred in patients with a known history of SLE. On the basis of the clinical presentation, we were unable to determine the temporal relationship between SLE and myelofibrosis, but we speculate that although the patient did not exhibit sufficient clinical evidence for the diagnosis of SLE upon initial presentation, the associated myelofibrosis worsened and improved with flares in SLE. In addition, the improvement of the patient's severe anemia with steroids alone also suggests an autoimmune cause.

Corticosteroids have been shown to improve the hematologic abnormalities observed in SLE.^3,4^ A review of literature conducted by Bass et al showed that the degree of bone marrow reticulin fibrosis did not change in approximately 50% of the cases. However, they did report that in approximately 5 of 7 cases in which the degree of fibrosis did not change, the patient's blood parameters improved. Near complete or complete resolution of fibrosis was observed in only 4 of 15 of the patients evaluated. Some reports have shown an improvement in the degree of myelofibrosis and resolution of pancytopenia after treatment with steroids.^13^ In our patient, the bone marrow did not improve much after treatment with corticosteroids. This may be because the grade of fibrosis is a rough measurement and bone marrow fibrosis resolution may take years. It is possible that an improvement in our patient's bone marrow will be observed at future examinations.

As demonstrated in our case, it is imperative that myelofibrosis be considered as a possible presentation of SLE, and that SLE be considered in the differential diagnosis of myelofibrosis. Furthermore, myelofibrosis in the setting of SLE is potentially reversible with corticosteroids, which would thus spare patients from unnecessary bone marrow transplantations. Prior literature and our case demonstrate the importance of considering autoimmune diseases when a patient presents with bone marrow myelofibrosis, and our case serves as a critical reminder of the several ways in which SLE can present.

We conclude by recommending increased awareness of this distinct clinicopathologic condition to increase knowledge of this rare but potentially life-threatening condition. Careful clinical assessment and bone marrow morphologic analysis is required. Anemia may be the only presenting sign initially, along with bone marrow fibrosis, and patients with autoimmune myelofibrosis can benefit from corticosteroid therapy.
